# Healthy Vision Month — May 2019

**DOI:** 10.15585/mmwr.mm6820a1

**Published:** 2019-05-24

May is Healthy Vision Month, an annual observance dedicated to making vision and eye health a national priority. During this month, CDC’s Vision Health Initiative (VHI) in the Division of Diabetes Translation joins with the National Eye Institute’s National Eye Health Education Program to educate the public about preventing vision loss and promoting eye health. Almost 3.22 million U.S. persons are affected by vision impairment, which can be associated with social isolation, disability, and decreased quality of life (https://www.cdc.gov/visionhealth/risk/burden.htm).

In this issue of *MMWR*, VHI staff members report findings from their study examining the association of vision impairment and functional limitations related to subjective cognitive decline (SCD), defined as the experience of worsening or more frequent confusion or memory loss (*1*). Analysis of data from the Behavioral Risk Factor Surveillance System survey for the years 2015–2017 indicated that persons with vision impairment were 3.5 times more likely to report functional limitations related to SCD than were those with no vision impairment.

With the number of U.S. adults with vision impairment projected to double in the next 30 years (*2*), understanding the impact of comorbid vision and SCD on functioning is just one of many important public health concerns related to vision loss. For information on topics related to vision and eye health, including common eye disorders, prevention, and related state and community programs, please visit the VHI web page (https://www.cdc.gov/visionhealth).
